# Perceptions of Partner Cognitive Ability During the COVID-19 Pandemic

**DOI:** 10.1093/geroni/igab046.3721

**Published:** 2021-12-17

**Authors:** Celinda Reese-Melancon, Jennifer Margrett, Dan Russell, Rachael Turner, Erin Harrington, Lauren Stratton, Jyoti Savla

**Affiliations:** 1 Oklahoma State University, Oklahoma State University, Oklahoma, United States; 2 Iowa State University, Ames, Iowa, United States; 3 Oklahoma State University, Stillwater, Oklahoma, United States; 4 Pennsylvania State University, University Park, Pennsylvania, United States; 5 Virginia Tech, Blacksburg, Virginia, United States

## Abstract

Media reports provide anecdotal evidence of increased forgetfulness during the COVID-19 pandemic (Cushing, 2021; Purtill, 2020). Scientific evidence suggests social isolation can impact on cognition (Evans et al., 2018), but the question remains whether those living with a partner experience similar deficits. The present study examined whether middle-aged and older adults’ perceptions of their own and their partner’s memory abilities were related to self-reported impact of the pandemic on daily life (e.g., limited social interactions, delayed health care, and disruption to routine). In a sample of 80 married individuals (49% female; age range 40-86 years), we found that participants’ beliefs about the impact of the pandemic on daily life and their depression ratings significantly predicted (p<0.05) their perceptions of their partner’s prospective memory abilities. Specifically, pandemic impact on daily life predicted 9.3% of the variance in participants’ reported perceptions of their partners’ prospective memory abilities, and participant depression ratings predicted an additional 5.1% of the variance. Surprisingly, these variables did not predict perceptions of participants’ own cognition or perceptions of partners’ retrospective memory abilities. In sum, people who reported greater impact of the pandemic on their lives were more likely to believe that their partner frequently forgot to carryout prospective memory intentions (e.g., failed to pass along a message or take medication), and depression further clouded their perception of their partner’s cognition. These findings should be extended to consider relationship quality and whether individuals consider their partners a reliable source of external memory support during times of life disruption.

